# Lower left ventricular ejection time in *MYBPC3* variant carriers with overt or subclinical hypertrophic cardiomyopathy

**DOI:** 10.1002/ehf2.15346

**Published:** 2025-07-04

**Authors:** Isabell Yan, Zoe Möhring, Daniel Reichart, Fanny Kortüm, Julia Münch, Rixa Woitschach, Paulus Kirchhof, Lucie Carrier, Carolyn Y. Ho, Thomas Eschenhagen, Monica Patten

**Affiliations:** ^1^ Department of Cardiology University Heart and Vascular Center Hamburg, University Medical Center Hamburg‐Eppendorf Hamburg Germany; ^2^ Cardiovascular Division Brigham and Women's Hospital Boston Massachusetts USA; ^3^ Department of Medicine I University Hospital, Ludwig Maximilian University of Munich Munich Germany; ^4^ Institute of Human Genetics University Medical Center Hamburg‐Eppendorf Hamburg Germany; ^5^ German Centre for Cardiovascular Research (DZHK), Partner Site Hamburg/Kiel/Lübeck Hamburg Germany; ^6^ Institute of Experimental Pharmacology and Toxicology University Medical Center Hamburg‐Eppendorf Hamburg Germany

**Keywords:** genetic variant, hypertrophic cardiomyopathy, left ventricular ejection time, *MYBPC3*, *MYH7*

## Abstract

**Aims:**

Hypertrophic cardiomyopathy (HCM) is an inherited cardiomyopathy often caused by pathogenic variants in *MYBPC3* and *MYH7*, encoding myosin‐binding protein C3 and myosin heavy chain 7, respectively. These variants can cause increased actin–myosin crossbridge cycling, resulting in ventricular hypercontractility, but mice lacking *Mybpc3* exhibited reduced left ventricular ejection time (LVET) as a sign of systolic dysfunction. In this study, we tested whether LVET is specifically altered in patients carrying *MYBPC3* variants by retrospective echocardiographic analysis in two genotype‐defined HCM cohorts.

**Methods:**

LVET was measured by echocardiography and adjusted for heart rate [LVET index (LVETI)] in 166 patients. Variant carriers were stratified for the presence (LVH+) or absence of left ventricular hypertrophy with septal thickness of ≥13 mm (LVH−). Multivariate analysis of variance (MANOVA) was used to identify differences in LVETI between variant carriers and controls with LVETI as the dependent variable, adjusted for sex, age, left ventricular ejection fraction (LVEF), interventricular septal diameter in diastole (IVSd), diastolic dysfunction, left ventricular outflow tract (LVOT) gradient at rest and medication history as confounders.

**Results:**

In a total of 166 patients carrying *MYBPC3* or *MYH7* pathogenic variants (38 ± 3 years, 45% female), we compared the discovery cohort (40 *MYBPC3* and 31 *MYH7*) and the validation cohort (‘Valsartan in Attenuating Disease Evolution in Early Sarcomeric HCM’; 54 *MYBPC3* and 41 *MYH7*) with 44 healthy controls. LVETI was lower in *MYBPC3* and higher in *MYH7* LVH+ patients than in controls in the discovery, validation and pooled cohorts (pooled: *MYBPC3* 381 ± 19 ms vs. *MYH7* 437 ± 38 ms, *P* < 0.001; *MYBPC3* vs. controls 411 ± 15 ms, *P* < 0.001; and *MYH7* vs. controls, *P* < 0.001). Similar findings were seen in LVH− (pooled: *MYBPC3* 380 ± 16 ms vs. *MYH7* 437 ± 39 ms, *P* < 0.001; *MYBPC3* vs. controls, *P* < 0.001). While *MYH7* variants were all missense as expected, 87% of the *MYBPC3* variants were truncating (including nonsense variants, out‐of‐frame deletion and splice site variants) and 13% were non‐truncating (missense and in‐frame deletion). LVETI did not differ between the groups and was significantly lower than the control in both.

**Conclusions:**

The data suggest that variants in *MYBPC3* and *MYH7* result in distinct biophysical consequences, which can be detected by measuring LVETI in patients. The findings may have implications for potential genotype‐specific differences in response to therapies targeting sarcomere function.

## Introduction

Hypertrophic cardiomyopathy (HCM) is characterized by thickening of the left ventricular (LV) wall, contractile abnormalities and potentially fatal arrhythmias. It is often caused by variants in genes such as *MYBPC3* and *MYH7*, encoding myosin‐binding protein C3 and myosin heavy chain 7, respectively.^1^ Pathophysiological consequences are altered sarcomere function leading to prolonged relaxation kinetics, oxidative and metabolic stress, and hyperdynamic contractility.^2^, ^3^, ^4^

The clinical presentation of patients with HCM carrying *MYH7* or *MYBPC3* variants appears similar by standard imaging techniques.^5^ HCM arising from either disease gene is associated with a broad range of clinical manifestations, from asymptomatic to advanced heart failure and sudden cardiac death. However, the consequences of variants in *MYH7* and *MYBPC3* differ at the molecular level. Pathogenic variants in *MYH7* are mainly missense, resulting in abnormal proteins that interfere with normal sarcomere function.^6^ In contrast, pathogenic *MYBPC3* variants, which account for the majority of genetic HCM cases, are typically truncating, leading to aberrant splicing and reduction of total MYBPC3 protein abundance (haploinsufficiency).^7^ Homozygous *Mybpc3* knock‐out mice present with dilated cardiomyopathy and LV hypertrophy^8^ and strongly reduced LV ejection time (LVET) index (LVETI). Such a phenotype was not found in four other mouse models of systolic dysfunction.^9^ This phenotype is supported by reduced contraction time in engineered heart tissue from *Mybpc3* knock‐out mice^10^, ^11^ or mice with biallelic *MYBPC3* mutations.^12^

LVETI reflects the duration of contraction and is a marker of LV contractile function, distinct from peak force, reflected by LV ejection fraction (LVEF). It is not yet used in routine clinical testing but has gained interest as an indicator of inotropic drug action. LVET is also considered a direct measure of stroke volume,^13^ and reduced LVETI was shown to be an independent predictor for heart failure and mortality.^14^

Here, we aimed to test the hypothesis that reduced LVETI may be a specific alteration in *MYBPC3*‐related HCM by performing echocardiographic analysis of genotyped cohorts of patients with HCM.

## Methods

### Cohort characteristics

**Figure 1:**
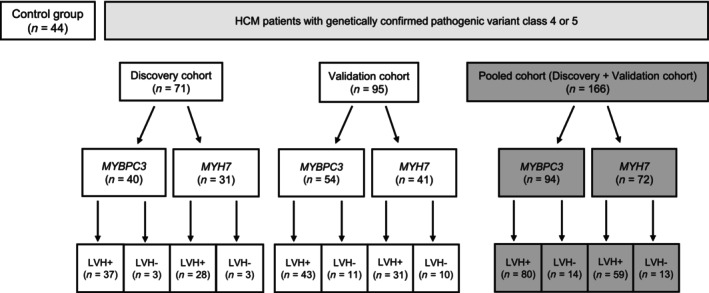
Graphic overview of the cohorts. Control group = healthy controls of the Hamburg cohort from the MOVE Study^15^; pathogenic variant Class 4 or 5 = according to the American College of Medical Genetics and Genomics^18^ standards and guidelines. LVH−, septum thickness <13 mm; LVH+, septum thickness ≥13 mm.

LVETI was first retrospectively determined in patients with HCM seen at the outpatient clinic of the University Heart and Vascular Center Hamburg. They were compared with data from age‐ and sex‐matched healthy controls of the Hamburg cohort from the MOVE Study (Figure 1).^15^ The results were validated in participants of the ‘Valsartan in Attenuating Disease Evolution in Early Sarcomeric HCM’ (VANISH) trial.^16^ In the pooled cohort, 166 patients with HCM had a genetically confirmed pathogenic variant Class 4 or 5 (94 *MYBPC3* and 72 *MYH7*). One hundred thirty‐nine of the patients had a phenotype with an interventricular septal diameter in diastole (IVSd ≥ 13 mm, LVH+). Twenty‐seven patients carrying variant Class 4 or 5 had a subclinical phenotype (IVSd < 13 mm, LVH−). A cutoff value of interventricular septum thickness (IVS) = 13 mm was used according to current recommendations for HCM classification.^17^

In total, 17 patients (8 *MYH7* and 9 *MYBPC3*) had arterial hypertension; all of them received pharmacological treatment. Twenty‐two patients (10 *MYH7* and 12 *MYBPC3*) had atrial fibrillation in their history. None were diagnosed with aortic stenosis. Elevated LV outflow tract (LVOT) gradients >30 mmHg were detected in three patients at rest (two *MYH7* and one *MYBPC3*), in two patients under Valsalva (two *MYH7*) and in two patients in ergometric stress echocardiography (one *MYH7* and one *MYBPC3*). Fifteen patients received septal reduction therapy: 12 by myectomy (6 *MYH7* and 6 *MYBPC3*) and 3 by alcohol septal ablation (3 *MYBPC3*).

LVET (defined as the time from opening of the aortic valve to its closure) was measured from transthoracic echocardiography by two independent investigators, who were blinded to genotype. Because LVET is inversely related to heart rate (HR), LVETI was used assuming a linear relationship between LVET and HR. The formula was derived on the basis of sex‐specific resting regression equations (for female: LVETI = 1.6 × HR + LVET; for male: LVETI = 1.7 × HR + LVET). Normal values for LVETI are 413 ± 10 ms in males and 418 ± 10 ms in females.^19^ Analyses of genetic variants and LVETI were adjusted for other disease‐specific parameters (LVEF, IVSd and LVOT gradient) and for other clinical characteristics (age, sex and concomitant medication use). Analyses were performed in LVH+ and LVH− groups.

### Genetic methods

In the discovery cohort, DNA sequencing was performed using next‐generation sequencing (NGS) after targeted enrichment (all kits provided by Illumina®). Library preparation and enrichment of target regions were conducted using the DNA Prep with Enrichment Kit and the TruSight™ Cardio Sequencing Kit, respectively. Subsequently, clustering and 150 bp paired‐end sequencing were performed on an Illumina® sequencing platform. After processing raw sequence data, Sequence Pilot software module SeqNext (JSI Medical Systems GmbH) was used for analysis of all coding as well as flanking non‐coding regions (−20/+10 bp) of the genes mentioned below (human genome build 37/hg19). Regions covered <20‐fold were additionally analysed by Sanger sequencing (corresponding to 100% coverage). Copy number analysis from the targeted NGS data was performed using the *Sequence Pilot module SeqNext* software (except for *TTN*). Potential copy number changes were validated by multiplex ligation‐dependent probe amplification (MLPA), a multiplex PCR method allowing for the identification of exonic copy number alterations (i.e., deletions and duplications), if a specific kit was available.

In the validation cohort VANISH, participants were required to carry HCM sarcomeric variants. Variant pathogenicity was determined using standard criteria accounting for segregation, conservation, published information and public databases, and absence or very low frequency in appropriate control populations. Investigators with expertise in genetics reviewed questionable variants to determine eligibility by consensus.^16^

Interpretation of variants was performed according to American College of Medical Genetics and Genomics (ACMG)^18^ standards and guidelines. Classification of variants was performed according to the five‐tier terminology system of the International Agency for Research on Cancer (IARC). Reporting of variants followed the most recent Human Genome Variation Society (HGVS) nomenclature guidelines. Used in silico prediction tools were CADD, Revel, M‐CAP, PolyPhen‐2 and Alamut (Sophia Genetics).

### Genetic characteristics

In patients with *MYBPC3* variants, ~87% were truncating (including nonsense variants, out‐of‐frame deletions and splice site variants; Table 1), expected to result in a loss of protein; 11% were missense variants, and 2% were in‐frame‐deletions, expected to result in stable but dysfunctional protein. In patients with *MYH7* variants, all variants were missense variants; 56 out of 72 (78%) were located in the head S1 domain, 8 out of 72 (11%) in the lever arm S2 domain and 8 out of 72 (11%) in the tail light meromyosin (LMM) domain. Out of the *MYBPC3* variants, 9 (10%) were classified as ACMG4 and 85 (90%) as ACMG5. Out of the *MYH7* variants, 17 (24%) were ACMG4 and 55 (76%) were ACMG5.

**Table 1:** Distribution of *MYBPC3* variants and *MYH7* variants.

MYBPC3			
	Truncating	Missense	IFD
Discovery cohort			
40	34	5	1
	0.85	0.12	0.03
Validation cohort			
54	48	5	1
	0.89	0.09	0.02
Pooled cohort			
94	82	10	2
	0.87	0.11	0.02

MYH7			
	Missense S1 domain	Missense S2 domain	Missense LMM
Discovery cohort			
31	23	2	6
	0.74	0.06	0.2
Validation cohort			
41	33	6	2
	0.8	0.15	0.05
Pooled cohort			
72	56	8	8
	0.77	0.11	0.11

*Note*: Different genotypes of *MYBPC3* variants are shown. It is divided into three groups: truncating variants (including nonsense variants, out‐of‐frame deletions and splice site variants), missense variants and IFD. *MYH7* variants are divided in S1 domain, S2 domain and LMM. S1 domain spans from Amino Acids 1–837, S2 domain spans from Amino Acids 838–1371, and then LMM is the rest.

Abbreviations: IFD, in‐frame deletion; LMM, light meromyosin.

### Statistics

The power calculation assumed an LVETI difference of 12 ms between the control group and patients with HCM mutation carriers to be clinically relevant, based on a study on patients with diastolic dysfunction.^19^, ^20^ It resulted in a minimum sample size of *n* = 18 and *n* = 36 for single and combined cohorts, respectively, at a desired *P* value of 0.05 and a power of 95%. For continuous variables (LVETI, age, LVEF, IVSd, LVOT gradient at rest and LVOT gradient during exercise), the mean value and standard deviation (SD) were specified. The nominal data (gender and medication intake) were presented using absolute and relative proportions (number and percentage). Analyses were performed comparing *MYH7* and *MYBPC3* variant carriers with either overt or subclinical HCM (LVH+ and LVH−). An unpaired Student's *t* test was used to compare continuous variables.

A multivariate analysis of variance (MANOVA) was used to identify differences in LVETI in variant carriers and controls with LVETI as the dependent variable, adjusted for sex, age, LVEF, IVSd, diastolic dysfunction, LVOT gradient at rest and medication history as confounders. To analyse the pattern of differences of means between two groups, we used Fisher's least significant difference (LSD) method as a post hoc test to perform the pairwise comparisons. The stored residuals were visually evaluated for normal distribution. All analyses were performed with *IBM SPSS Statistics*; a *P* value <0.05 was considered statistically significant. Box plots were generated with GraphPad Prism Version 5.

## Results

### Discovery cohort

The discovery cohort from Hamburg comprised 40 patients with HCM carrying *MYBPC3* variants and 31 with *MYH7* variants (Table 2 and Figure 1). The mean age was 53 and 47 years, respectively. The mean age of controls was 44 years. The percentage of women was 55%, 64% and 50% in the *MYBPC3*, *MYH7* and control groups, respectively. Healthy controls had an LVETI of 411 ± 15 ms, patients with *MYBPC3* variants had 387 ± 18 ms (*P* = 0.004 vs. controls), and patients with *MYH7* had 445 ± 33 ms (*P* < 0.001 vs. *MYBPC3* and *P* = 0.03 vs. controls).

**Table 2:** Clinical characteristics of patients with HCM and controls.

		*MYH7* (*n* = 72)						*MYBPC3* (*n* = 94)					
		LVH− (*n* = 13)			LVH+ (*n* = 59)			LVH− (*n* = 14)			LVH+ (*n* = 80)		
	Control (*n* = 44)	Hamburg (*n* = 3)	VANISH (*n* = 10)	*P* value	Hamburg (*n* = 28)	VANISH (*n* = 31)	*P* value	Hamburg (*n* = 3)	VANISH (*n* = 11)	*P* value	Hamburg (*n* = 37)	VANISH (*n* = 43)	*P* value
Age (years), mean (SD); [95% CI]	44 (12); [40–47]	26 (3); [19–34]	27 (7); [21–32]	0.954	47 (15); [41–53]	30 (8); [27–33]	<0.001	37 (14); [3–72]	21 (4); [19–24]	0.176	51 (15); [45–56]	30 (8); [27–32]	<0.001
Female sex, *n* (%)	22 (50)	1 (33)	8 (80)	0.125	18 (64)	12 (39)	0.05	2 (67)	4 (36)	0.347	16 (43)	13 (30)	0.227
LVEF (%), mean (SD); [95% CI]	62 (4); [61–63]	57 (1); [55–59]	59 (2); [58–61]	0.09	56 (3); [55–57]	59 (3); [57–60]	0.004	57 (2); [53–60]	60 (3); [58–62]	0.07	55 (7); [53–58]	60 (3); [59–61]	0.001
IVSd (mm), mean (SD); [95% CI]	9 (1); [9–10]	10 (2); [7–14]	11 (1); [10–12]	0.484	22 (5); [20–24]	18 (4); [16–19]	<0.001	12 (1); [10–13]	11 (2); [9–12]	0.26	21 (6); [19–23]	17 (4); [15–18]	<0.001
LVOT gradient rest (mmHg), mean (SD); [95% CI]	<5	6 (2); [1–12]	6 (1); [5–6]	0.6	11 (9); [8–15]	6 (2); [5–7]	0.004	6 (1); [4–7]	5 (1); [4–6]	0.339	11 (13); [6–15]	7 (6); [5–9]	0.175
LVOT gradient max (mmHg), mean (SD); [95% CI]	<5	6 (2); [1–12]	‐	‐	22 (24); [13–32]	‐	‐	6 (1); [4–7]	‐	‐	16 (21); [9–23]	‐	‐
ACE inhibitor, *n* (%)	0 (0)	1 (33)	0 (0)	0.057	2 (7)	0 (0)	0.13	0 (0)	0 (0)	‐	7 (19)	0 (0)	0.003
Beta‐blocker, *n* (%)	0 (0)	0 (0)	0 (0)	‐	19 (68)	10 (32)	0.006	0 (0)	2 (18)	0.425	28 (76)	8 (19)	<0.001
Calcium channel blockers, *n* (%)	0 (0)	1 (33)	0 (0)	0.057	1 (4)	2 (7)	0.615	0 (0)	1 (9)	0.588	4 (11)	1 (2)	0.118
Diastolic dysfunction, *n* (%)	7 (16)	0 (0)	2 (20)	0.4	17 (61)	25 (81)	0.091	0 (0)	0 (0)	‐	19 (51)	30 (70)	0.092

*Note*: The patients were separated in groups with overt HCM (LVH+, IVSd ≥ 13 mm) or subclinical HCM (LVH−, IVSd < 13 mm) and compared with a control cohort from Hamburg, Germany. A *P* value <0.05 was considered statistically significant.

Abbreviations: ACE, angiotensin‐converting enzyme; CI, confidence interval; HCM, hypertrophic cardiomyopathy; IVSd, interventricular septal diameter in diastole; LVEF, left ventricular ejection fraction; LVH, left ventricular hypertrophy; LVOT, left ventricular outflow tract; *MYBPC3*, myosin‐binding protein C3 gene; *MYH7*, myosin heavy chain 7 gene; *n*, number of individuals; VANISH, Valsartan in Attenuating Disease Evolution in Early Sarcomeric HCM.

**Figure 2:**
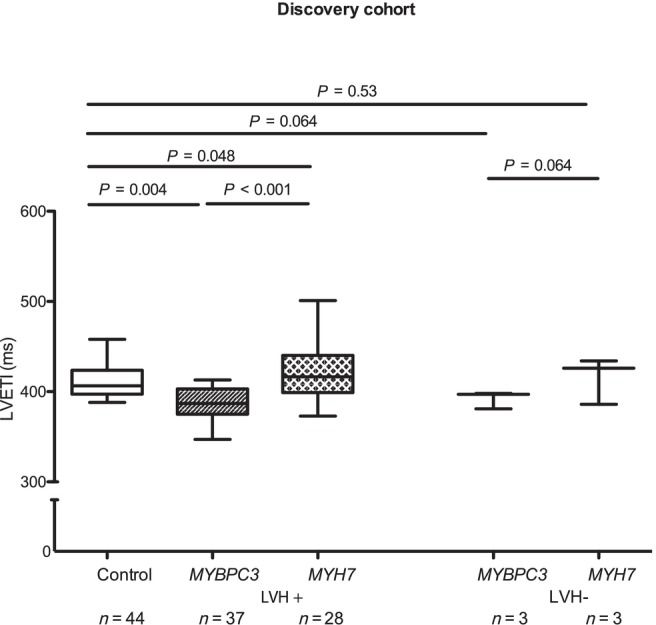
Left ventricular ejection time index (LVETI) in healthy controls and hypertrophic cardiomyopathy (HCM) patients in the discovery cohort. The patients were separated in groups with overt HCM [LVH+, interventricular septal diameter in diastole (IVSd) ≥13 mm] or subclinical HCM (LVH−, IVSd < 13 mm). The statistical significance was assessed by multivariate analysis of variance. A linear model was used for comparison between groups, adjusted for age, sex, left ventricular ejection fraction, IVSd and gradient at rest. Data are shown as median and 1.5 inter‐quartile range. LVH, left ventricular hypertrophy; *MYBPC3*, myosin‐binding protein C3 gene; *MYH7*, myosin heavy chain 7 gene; *n*, number of individuals. A *P* value <0.05 was considered statistically significant.

Subgroup analyses were performed on LVH+ patients (37 *MYBPC3* and 28 *MYH7*) and LVH− patients (3 *MYBPC3* and 3 *MYH7*). *MYBPC3* LVH+ patients had a significantly lower LVETI than controls (387 ± 19 vs. 411 ± 15 ms; *P* = 0.004) and *MYH7* LVH+ patients (437 ± 38 ms; *P* < 0.001 vs. *MYBPC3*). LVETI was also lower in *MYBPC3* LVH− than in *MYH7* LVH− patients (392 ± 10 vs. 425 ± 9 ms; *P* = 0.064; Figure 2).

### Validation cohort

The validation cohort from the VANISH trial comprised 54 patients carrying *MYBPC3* variants and 41 patients carrying *MYH7* variants. The mean age was 28 and 29 years, respectively. The percentage of women was 32% and 39% in the *MYBPC3* and *MYH7* groups, respectively. Patients with *MYBPC3* variants had an LVETI of 376 ± 17 ms (*P* < 0.001 vs. controls) and *MYH7* carriers an LVETI of 447 ± 39 ms (*P* < 0.001 vs. *MYBPC3* and control groups).

**Figure 3:**
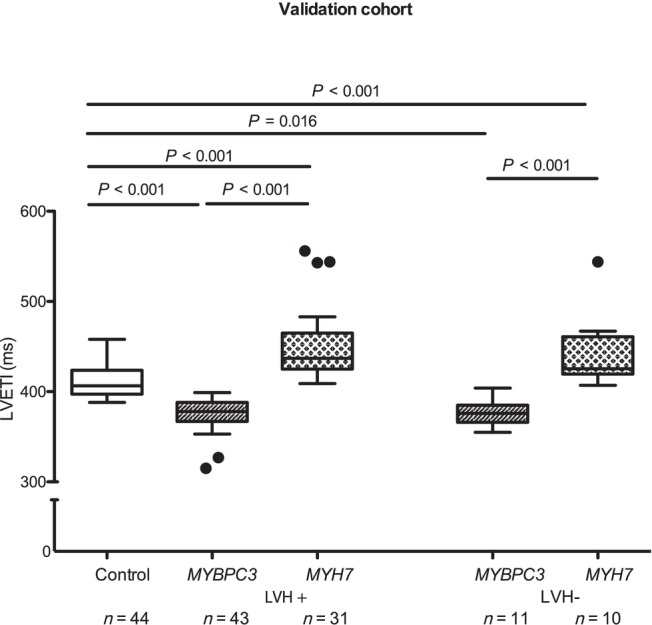
Left ventricular ejection time index (LVETI) in healthy controls and hypertrophic cardiomyopathy (HCM) patients in the validation cohort. The participants of the VANISH trial were separated in groups with overt HCM [LVH+, interventricular septal diameter in diastole (IVSd) ≥13 mm] or subclinical HCM (LVH−, IVSd < 13 mm). The statistical significance was assessed by multivariate analysis of variance. A linear model was used for comparison between groups, adjusted for age, sex, left ventricular ejection fraction, IVSd and gradient at rest. Data are shown as median and 1.5 inter‐quartile range. LVH, left ventricular hypertrophy; *MYBPC3*, myosin‐binding protein C3 gene; *MYH7*, myosin heavy chain 7 gene; *n*, number of individuals. A *P* value <0.05 was considered statistically significant.

In the subgroup analysis, LVETI was lower in *MYBPC3* LVH+ than in both controls (376 ± 17 vs. 411 ± 15 ms; *P* < 0.001) and *MYH7* LVH+ (449 ± 39 ms; *P* < 0.001 vs. *MYBPC3*; Figure 2). Similarly, LVETI was lower in *MYBPC3* LVH− (377 ± 16 ms) than in *MYH7* LVH− (443 ± 40 ms; *P* < 0.001; Figure 3).

### Pooled cohort

Combining the discovery and validation cohorts, patients with *MYBPC3* variants (*n* = 94) showed the shortest LVETI (381 ± 18 ms; *P* < 0.001 vs. control) and *MYH7* carriers (*n* = 72) the longest LVETI (436 ± 38 ms; *P* < 0.001 vs. *MYBPC3* and *P* = 0.003 vs. control groups).

**Figure 4:**
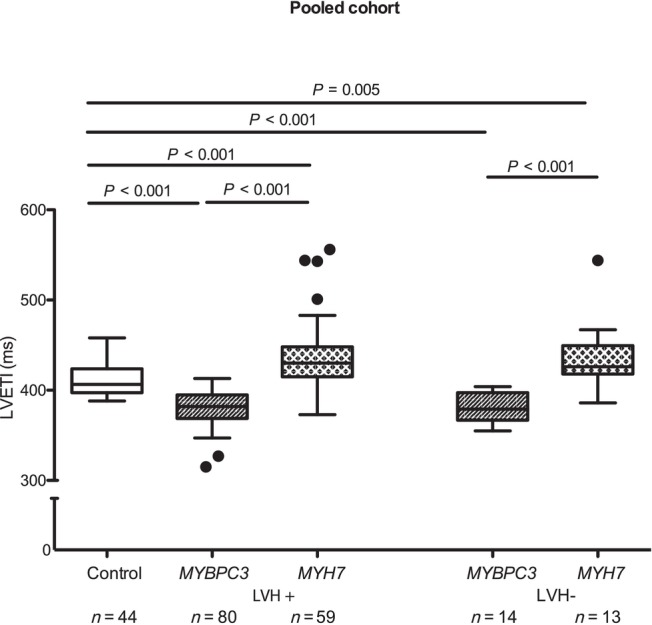
Left ventricular ejection time index (LVETI) in healthy controls and hypertrophic cardiomyopathy (HCM) patients in the pooled cohort. The patients were separated in groups with overt HCM [LVH+, interventricular septal diameter in diastole (IVSd) ≥13 mm] or subclinical HCM (LVH−, IVSd < 13 mm). The statistical significance was assessed by multivariate analysis of variance. A linear model was used for comparison between groups, adjusted for age, sex, left ventricular ejection fraction, IVSd and gradient at rest. Data are shown as median and 1.5 inter‐quartile range. LVH, left ventricular hypertrophy; *MYBPC3*, myosin‐binding protein C3 gene; *MYH7*, myosin heavy chain 7 gene; *n*, number of individuals. A *P* value <0.05 was considered statistically significant.

In the subgroup analysis, LVETI was lower in *MYBPC3* LVH+ (*n* = 80, 381 ± 19 ms) than in both *MYH7* LVH+ patients (*n* = 59, 437 ± 38 ms; *P* < 0.001) and controls (*n* = 44, 411 ± 15 ms; *P* < 0.001; Figure 3). LVETI was also lower in *MYBPC3* LVH− (*n* = 14, 380 ± 16 ms) than in *MYH7* LVH− (*n* = 13, 436 ± 39 ms; *P* < 0.001) and controls (*P* < 0.001 vs. *MYBPC3* and *P* < 0.001 vs. *MYH7*; Figure 4).

**Figure 5:**
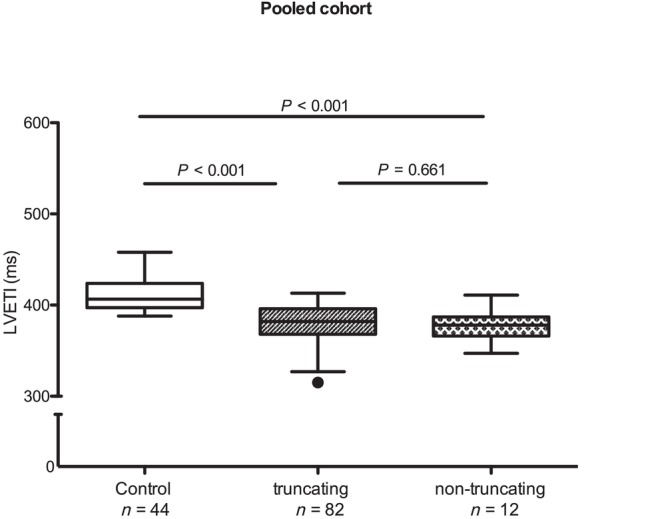
Left ventricular ejection time index (LVETI) in healthy controls and patients with truncating and non‐truncating myosin‐binding protein C3 gene (*MYBPC3*) variants in pooled cohort. The patients were separated in groups with truncating and non‐truncating variants. The statistical significance was assessed by multivariate analysis of variance. A linear model was used for comparison between groups, adjusted for age, sex, left ventricular ejection fraction, interventricular septal diameter in diastole and gradient at rest. Data are shown as median and 1.5 inter‐quartile range. LVH, left ventricular hypertrophy; *MYH7*, myosin heavy chain 7 gene; *n*, number of individuals. A *P* value <0.05 was considered statistically significant.

To evaluate whether the type of mutation had an influence on the results, we stratified the group in carriers of a truncating or non‐truncating variant (Table 1). While *MYH7* variants were all missense as expected, 87% of the *MYBPC3* variants were truncating (including nonsense variants, out‐of‐frame deletion and splice site variants) and 13% were non‐truncating (missense and in‐frame deletion). LVETI did not differ between the groups and was significantly lower than the control in both (Figure 5).

### Clinical characteristics and potential confounders

Some disease‐specific parameters such as LVEF, IVSd, diastolic dysfunction, LVOT gradient at rest and medication history did reveal statistically significant differences between the discovery and validation LVH+ groups (Table 2). There was a slightly lower ejection fraction (EF), a more pronounced IVSd and a higher LVOT gradient in the discovery than in the validation cohort. These differences align well with the older mean age in the discovery LVH+ group. LVEF was normal in both cohorts, septal thickness was severe in both cohorts, and the mean gradient in both cohorts was below 30 mmHg, indicating no obstruction at rest.

A MANOVA was used to identify differences in LVETI in variant carriers and controls with LVETI as the dependent variable, adjusted for sex, age, LVEF, IVSd, diastolic dysfunction, LVOT gradient at rest and medication history as confounders (Table 3). The analysis showed only differences in LVETI between the groups, but confounders as sex, age, LVEF, diastolic dysfunction, LVOT gradient at rest and medication revealed no significant influence on LVETI.

**Table 3:** Impact of potential confounders on LVETI.

Confounders	Discovery cohort *P* value	Validation cohort *P* value	Pooled cohort *P* value
Sex	0.75	0.51	0.74
Diastolic dysfunction	0.53	0.37	0.65
Age	0.66	0.19	0.69
LVEF	0.23	0.82	0.49
IVS	0.10	0.09	0.09
Gradient at rest	0.28	0.64	0.20
Beta‐blocker	0.97	0.15	0.10
ACE inhibitor	0.13	‐	0.47
Calcium channel blocker	0.79	0.77	0.80

*Note*: *P* values in the discovery cohort, validation cohort and pooled cohort are shown whether the confounders have a significant influence on LVETI.

Abbreviations: ACE, angiotensin‐converting enzyme; LVEF, left ventricular ejection fraction; LVETI, left ventricular ejection time index.

## Discussion

This study demonstrates distinct genotype‐related differences in the duration of cardiac contraction in patients with HCM. LVETI was significantly lower than in healthy controls in individuals with pathogenic *MYBPC3* variants and significantly higher in individuals with *MYH7* variants. The differences in mean LVETI (pooled cohort) between *MYBPC3* carriers and controls (−30 ms) and between *MYH7* carriers and controls (+26 ms), respectively, are larger than the reported difference between patients with diastolic dysfunction and controls (−12 ms).^19^, ^20^ Similarly, they were similar to or larger than those reported for patients with mildly, moderately and severely reduced EF (−4, −8 and −28 ms) compared with healthy controls.^21^ The size of the differences argues for their pathophysiological relevance. The differences in LVETI were observed not only in variant carriers with clinically overt HCM (LVH+) but also in LVH−, suggesting that alterations in ejection time are a primary manifestation of *MYBPC3* and *MYH7* genetic variations itself. This and the opposing direction of changes in LVETI in *MYBPC3* and *MYH7* variant carriers indicate that they are not a general consequence or feature of HCM. Confounders such as sex, age, LVEF, IVSd and gradient at rest showed no significant influence on LVETI. Medication with beta‐blockers and calcium channel blockers is the first‐line treatment in HCM. In our study, an effect of these drugs on LVETI could not be observed.

LVETI was lower in the *MYBPC3* than in the *MYH7* group despite similar degrees of cardiac hypertrophy, outflow tract obstruction and LVEF. This indicates that *MYBPC3* variants are indeed associated with a specific deficit in the late contraction/early relaxation phase as indicated by prior mouse and tissue studies.^1^, ^2^, ^9^, ^12^ Genetically induced homozygous reduction or loss of *MYBPC3* in mice led to reduced LVETI.^9^, ^12^ And human septal myectomy samples of patients with *MYBPC3*‐associated HCM showed higher rates of the fast‐exponential phase of relaxation.^22^ Additionally, shorter contraction time was seen in engineered heart tissues from *Mybpc3*‐targeted knock‐in mice.^10^, ^11^ These data support the hypothesis that MYBPC3 not only facilitates diastolic relaxation by promoting a super‐relaxed state of myosin but also stabilizes systole by binding to the thin filament.^9^ Reduction or loss of MYBPC3 may reduce the ability of the heart to maintain systolic ejection. In support of this hypothesis, Beltrami *et al*. recently showed in a retrospective analysis that *MYBPC3* variant carriers were more likely to develop systolic dysfunction over time than *MYH7* carriers.^23^ These data are somewhat at odds with earlier data suggesting a better survival prognosis of *MYBPC3* than *MYH7* carriers^24^ and certainly require validation. But it is in support of preclinical^9^, ^10^, ^11^, ^12^ and the present data, pointing at a specific systolic deficit conferred by *MYBPC3* variants. Our data indicate that such an effect is conferred by both truncating and non‐truncating *MYBPC3* variants, which was unexpected and is currently unexplained.

In contrast to routinely measured echocardiographic parameters such as LVEF and IVSd thickness, LVETI has not yet found its way into routine clinical practice. One reason may be that it is not altered in the general HCM cohort,^25^ which is understandable if one considers pathogenic *MYBPC3* and *MYH7* variants to have similar frequencies in HCM and have opposing effects on LVETI (this study). LVETI is increasingly recognized as an important metric of cardiac function and drug response in patients with heart failure and cardiomyopathies.^13^ As disease‐specific medications for HCM are beginning to enter clinical practice, including cardiac myosin inhibitors developed to slow cycling rate,^26^, ^27^ our findings may be relevant because they predict gene‐specific differences in the response to such therapies. A practical consequence of our findings could be a recommendation for early genotyping of patients with HCM. Alternatively, LVETI as an easy‐to‐measure echocardiographic parameter could be used to help select patients that most likely respond well to myosin inhibitors and genetic testing. Our data suggest, but do not prove, that patients with supranormal LVETI benefit more than patients with subnormal LVETI. LVETI may also be a surrogate parameter for adequate dosing of myosin inhibitors.

Further studies in larger cohorts are warranted to validate our results and to evaluate whether therapy responses in HCM may indeed differ in a genotype‐specific manner. Further correlation with advanced imaging parameters (e.g., myocardial strain, tissue characterization or late gadolinium enhancement) is a significant area for future research to clarify the relationship between ejection time and disease progression.

### Limitation

The main limitation of the study is the relatively small size of the cohort. Especially the LVH− group consists of only 27 patients, which may result in an underpowered analysis to detect significant differences. The control cohort was not genotyped, so it cannot be excluded that the control group may include some phenotype‐negative variant carriers. However, according to the extremely low prevalence of Class 4 and 5 variants in *MYBPC3* or *MYH7* in data banks such as gnomAD, the likelihood is very low. Another potential limitation is that the control group was only age‐matched to the discovery cohort; the average age of the validation group was younger. Cardiac markers or comorbidities could not be compared between the two HCM cohorts, as N‐terminal pro‐BNP (NT‐proBNP) and high‐sensitivity troponin I were measured in the control and discovery cohort, whereas BNP and troponin T were measured in the validation cohort. Data on comorbidities were only available in the discovery cohort.

## Conclusions

In conclusion, our study provides evidence for decreased duration of contraction in patients with HCM carrying *MYBPC3* variants. The reduced LVETI appears as a specific manifestation of *MYBPC3* gene variants, which can be determined by echocardiography even before a clinically overt disease develops. The findings may have implications for the natural history of disease as well as response to targeted therapies.

## Conflict of interest statement

P.K. received research support for basic, translational and clinical research projects from several drug and device companies active in atrial fibrillation and has received honoraria from several companies in the past, but not in the last 5 years. P.K. is listed as inventor on two issued patents held by the University of Hamburg (Atrial Fibrillation Therapy WO 2015140571 and Markers for Atrial Fibrillation WO 2016012783). L.C. and T.E. are advisors and shareholders of DiNAQOR AG developing an *MYBPC3*‐based gene therapy for HCM. C.Y.H. receives consulting honoraria from Bristol Myers Squibb, Pfizer, Cytokinetics, BioMarin Pharmaceutical, Tenaya, Viz.ai and Lexicon. M.P. receives consulting honoraria from Bristol Myers Squibb, Cytokinetics, Sanofi, Alnylam and Pfizer. I.Y., Z.M., D.R., F.K., J.M. and R.W. have nothing to disclose.

## Funding

P.K. is partially supported by grants from the European Union AFFECT‐AF (847770) and European Union MAESTRIA (965286), the British Heart Foundation (PG/20/22/35093 and AA/18/2/34218), the German Centre for Cardiovascular Research [Deutsches Zentrum für Herz‐Kreislaufforschung (DZHK); FKZ 81X2800182, 81Z0710116 and 81Z0710110], the German Ministry of Research Education [Bundesministerium für Bildung und Forschung (BMBF)] and the German Research Foundation (Deutsche Forschungsgemeinschaft; Ki 509167694). L.C. is supported by grants from the Leducq Foundation (Fondation Leducq; 20CVD01), the German Centre for Cardiovascular Research (DZHK) and the German Ministry of Research Education (BMBF). C.Y.H. is funded by the National Institutes of Health (USA) and receives unrestricted research funding from Bristol Myers Squibb, Pfizer, Cytokinetics, BioMarin Pharmaceutical, Tenaya, Viz.ai and Lexicon. The VANISH trial was funded by the National Heart, Lung, and Blood Institute (NHLBI) of the NIH (P50HL112349). T.E. is supported by grants from the German Research Foundation (DFG; Es 88/16‐1 and Es 88/17‐1) and the European Union's Horizon 2020 Research and Innovation Program (874764). M.P. receives funding from Takeda/Shire.

## References

[1] Frey N , Luedde M , Katus HA . Mechanisms of disease: Hypertrophic cardiomyopathy. Nat Rev Cardiol 2011;9:91‐100. doi:10.1038/nrcardio.2011.159 22027658

[2] Cohn R , Thakar K , Lowe A , Ladha FA , Pettinato AM , Romano R , *et al*. A contraction stress model of hypertrophic cardiomyopathy due to sarcomere mutations. Stem Cell Rep 2019;12:71‐83. doi:10.1016/j.stemcr.2018.11.015 PMC6335568 30554920

[3] Keren A , Syrris P , McKenna WJ . Hypertrophic cardiomyopathy: The genetic determinants of clinical disease expression. Nat Clin Pract Cardiovasc Med 2008;5:158‐168. doi:10.1038/ncpcardio1110 18227814

[4] Garfinkel AC , Seidman JG , Seidman CE . Genetic pathogenesis of hypertrophic and dilated cardiomyopathy. Heart Fail Clin 2018;14:139‐146. doi:10.1016/j.hfc.2017.12.004 29525643 PMC5851453

[5] Velicki L , Jakovljevic DG , Preveden A , Golubovic M , Bjelobrk M , Ilic A , *et al*. Genetic determinants of clinical phenotype in hypertrophic cardiomyopathy. BMC Cardiovasc Disord 2020;20:516. doi:10.1186/s12872-020-01807-4 33297970 PMC7727200

[6] Theis JL , Bos JM , Theis JD , Miller DV , Dearani JA , Schaff HV , *et al*. Expression patterns of cardiac myofilament proteins: Genomic and protein analysis of surgical myectomy tissue from patients with obstructive hypertrophic cardiomyopathy. Circ Heart Fail 2009;2:325‐333. doi:10.1161/CIRCHEARTFAILURE.108.789735 19808356 PMC2765062

[7] Tudurachi B‐S , Zăvoi A , Leonte A , Ţăpoi L , Ureche C , Bîrgoan SG , *et al*. An update on *MYBPC3* gene mutation in hypertrophic cardiomyopathy. Int J Mol Sci 2023;24:10510. doi:10.3390/ijms241310510 37445689 PMC10341819

[8] Carrier L . Asymmetric septal hypertrophy in heterozygous cMyBP‐C null mice. Cardiovasc Res 2004;63:293‐304. doi:10.1016/j.cardiores.2004.04.009 15249187

[9] Palmer BM , Georgakopoulos D , Janssen PM , Wang Y , Alpert NR , Belardi DF , *et al*. Role of cardiac myosin binding protein C in sustaining left ventricular systolic stiffening. Circ Res 2004;94:1249‐1255. doi:10.1161/01.RES.0000126898.95550.31 15059932

[10] de Lange WJ , Hegge LF , Grimes AC , Tong CW , Brost TM , Moss RL , *et al*. Neonatal mouse‐derived engineered cardiac tissue: A novel model system for studying genetic heart disease. Circ Res 2011;109:8‐19. doi:10.1161/CIRCRESAHA.111.242354 21566213 PMC3123426

[11] Stöhr A , Friedrich FW , Flenner F , Geertz B , Eder A , Schaaf S , *et al*. Contractile abnormalities and altered drug response in engineered heart tissue from *Mybpc3*‐targeted knock‐in mice. J Mol Cell Cardiol 2013;63:189‐198. doi:10.1016/j.yjmcc.2013.07.011 23896226

[12] Pietsch N , Chen CY , Kupsch S , Bacmeister L , Geertz B , Herrera‐Rivero M , *et al*. Chronic activation of tubulin tyrosination improves heart function. Circ Res 2024; doi:10.1161/CIRCRESAHA.124.324387 PMC11465905 39279670

[13] Alhakak AS , Teerlink JR , Lindenfeld J , Böhm M , Rosano GMC , Biering‐Sørensen T . The significance of left ventricular ejection time in heart failure with reduced ejection fraction. Eur J Heart Fail 2021;23:541‐551. doi:10.1002/ejhf.2125 33590579

[14] Biering‐Sørensen T , Querejeta Roca G , Hegde SM , Shah AM , Claggett B , Mosley TH , *et al*. Left ventricular ejection time is an independent predictor of incident heart failure in a community‐based cohort. Eur J Heart Fail 2018;20:1106‐1114. doi:10.1002/ejhf.928 28872225 PMC6685547

[15] Tahir E , Starekova J , Muellerleile K , von Stritzky A , Münch J , Avanesov M , *et al*. Myocardial fibrosis in competitive triathletes detected by contrast‐enhanced CMR correlates with exercise‐induced hypertension and competition history. JACC Cardiovasc Imaging 2018;11:1260‐1270. doi:10.1016/j.jcmg.2017.09.016 29248656

[16] Ho CY , Day SM , Axelsson A , Russell MW , Zahka K , Lever HM , *et al*. Valsartan in early‐stage hypertrophic cardiomyopathy: A randomized phase 2 trial. Nat Med 2021;27:1818‐1824. doi:10.1038/s41591-021-01505-4 34556856 PMC8666141

[17] Arbelo E , Protonotarios A , Gimeno JR , Arbustini E , Barriales‐Villa R , Basso C , *et al*. 2023 ESC guidelines for the management of cardiomyopathies: Developed by the Task Force on the Management of Cardiomyopathies of the European Society of Cardiology (ESC). Eur Heart J 2023;44:3503‐3626. doi:10.1093/eurheartj/ehad194 37622657

[18] Richards S , Aziz N , Bale S , Bick D , Das S , Gastier‐Foster J , *et al*. Standards and guidelines for the interpretation of sequence variants: A joint consensus recommendation of the American College of Medical Genetics and Genomics and the Association for Molecular Pathology. Genet Med 2015;17:405‐424. doi:10.1038/gim.2015.30 25741868 PMC4544753

[19] Weissler AM , Harris WS , Schoenfeld CD . Systolic time intervals in heart failure in man. Circulation 1968;37:149‐159. doi:10.1161/01.CIR.37.2.149 5640345

[20] Weber T , Auer J , O'Rourke MF , Punzengruber C , Kvas E , Eber B . Prolonged mechanical systole and increased arterial wave reflections in diastolic dysfunction. Heart 2006;92:1616‐1622. doi:10.1136/hrt.2005.084145 16709696 PMC1861240

[21] Haiden A , Eber B , Weber T . U‐shaped relationship of left ventricular ejection time index and all‐cause mortality. Am J Hypertens 2014;27:702‐709. doi:10.1093/ajh/hpt185 24108863

[22] Pioner JM , Vitale G , Steczina S , Langione M , Margara F , Santini L , *et al*. Slower calcium handling balances faster cross‐bridge cycling in human *MYBPC3* HCM. Circ Res 2023;132:628‐644. doi:10.1161/CIRCRESAHA.122.321956 36744470 PMC9977265

[23] Beltrami M , Fedele E , Fumagalli C , Mazzarotto F , Girolami F , Ferrantini C , *et al*. Long‐term prevalence of systolic dysfunction in *MYBPC3* versus *MYH7*‐related hypertrophic cardiomyopathy. Circ Genom Precis Med 2023;16:363‐371. doi:10.1161/CIRCGEN.122.003832 37409452

[24] Charron P , Dubourg O , Desnos M , Bennaceur M , Carrier L , Camproux AC , *et al*. Clinical features and prognostic implications of familial hypertrophic cardiomyopathy related to the cardiac myosin‐binding protein C gene. Circulation 1998;97:2230‐2236. doi:10.1161/01.cir.97.22.2230 9631872

[25] Bonow RO , Rosing DR , Bacharach SL , Green MV , Kent KM , Lipson LC , *et al*. Effects of verapamil on left ventricular systolic function and diastolic filling in patients with hypertrophic cardiomyopathy. Circulation 1981;64:787‐796. doi:10.1161/01.cir.64.4.787 7196813

[26] Maron MS , Masri A , Nassif ME , Barriales‐Villa R , Arad M , Cardim N , *et al*. Aficamten for symptomatic obstructive hypertrophic cardiomyopathy. N Engl J Med 2024;390:1849‐1861. doi:10.1056/NEJMoa2401424 38739079

[27] Olivotto I , Oreziak A , Barriales‐Villa R , Abraham TP , Masri A , Garcia‐Pavia P , *et al*. Mavacamten for treatment of symptomatic obstructive hypertrophic cardiomyopathy (EXPLORER‐HCM): A randomised, double‐blind, placebo‐controlled, phase 3 trial. The Lancet 2020;396:759‐769. doi:10.1016/S0140-6736(20)31792-X 32871100

